# Unique Effects of Acute Aripiprazole Treatment on the Dopamine D2 Receptor Downstream cAMP-PKA and Akt-GSK3β Signalling Pathways in Rats

**DOI:** 10.1371/journal.pone.0132722

**Published:** 2015-07-10

**Authors:** Bo Pan, Jiezhong Chen, Jiamei Lian, Xu-Feng Huang, Chao Deng

**Affiliations:** 1 Antipsychotic Research Laboratory, Illawarra Health and Medical Research Institute, Wollongong, NSW, Australia; 2 Centre for Translational Neuroscience, School of Medicine, University of Wollongong, Wollongong, NSW, Australia; UTHSCSH, UNITED STATES

## Abstract

Aripiprazole is a wide-used antipsychotic drug with therapeutic effects on both positive and negative symptoms of schizophrenia, and reduced side-effects. Although aripiprazole was developed as a dopamine D_2_ receptor (D_2_R) partial agonist, all other D_2_R partial agonists that aimed to mimic aripiprazole failed to exert therapeutic effects in clinic. The present *in vivo* study aimed to investigate the effects of aripiprazole on the D_2_R downstream cAMP-PKA and Akt-GSK3β signalling pathways in comparison with a D_2_R antagonist – haloperidol and a D_2_R partial agonist – bifeprunox. Rats were injected once with aripiprazole (0.75mg/kg, i.p.), bifeprunox (0.8mg/kg, i.p.), haloperidol (0.1mg/kg, i.p.) or vehicle. Five brain regions – the prefrontal cortex (PFC), nucleus accumbens (NAc), caudate putamen (CPu), ventral tegmental area (VTA) and substantia nigra (SN) were collected. The protein levels of PKA, Akt and GSK3β were measured by Western Blotting; the cAMP levels were examined by ELISA tests. The results showed that aripiprazole presented similar acute effects on PKA expression to haloperidol, but not bifeprunox, in the CPU and VTA. Additionally, aripiprazole was able to increase the phosphorylation of GSK3β in the PFC, NAc, CPu and SN, respectively, which cannot be achieved by bifeprunox and haloperidol. These results suggested that acute treatment of aripiprazole had differential effects on the cAMP-PKA and Akt-GSK3β signalling pathways from haloperidol and bifeprunox in these brain areas. This study further indicated that, by comparison with bifeprunox, the unique pharmacological profile of aripiprazole may be attributed to the relatively lower intrinsic activity at D_2_R.

## Introduction

Aripiprazole has therapeutic effects on both positive and negative symptoms of schizophrenia, with improved extrapyramidal side-effects (EPS) compared with first-generation antipsychotic drugs (e.g. haloperidol) and reduced metabolic side-effects compared with second-generation antipsychotic drugs (e.g. olanzapine); aripiprazole is regarded as the third-generation antipsychotic drug [1, 2]. The exact mechanisms of aripiprazole remain unclear. Several studies suggested that the potent partial agonism of aripiprazole for the dopamine D_2_ receptor (D_2_R) stabilises the dopamine D_2_ system, playing a critical role in its unique clinical actions [3, 4]. However, this hypothesis has been questioned because there are no other D_2_R partial agonists that are widely accepted and used after aripiprazole. On the other hand, a theory called functional selectivity has been postulated to explain the pharmacological profile of aripiprazole [1]. The theory of functional selectivity suggests that depending on the cellular location and environment of the target G protein-coupled receptors (GPCRs), some ligands (drugs) can induce distinct conformations of GPCRs, resulting in differential regulation of canonical and non-canonical signal transduction pathways associated with these GPCRs [5–7]. Accumulated evidence from *in vitro* studies suggested that aripiprazole displays functional selectivity on D_2_R; it may act as a potent partial agonist, weak agonist, or antagonist depending on the targeted D_2_Rs [1, 4, 6, 8–10]. An *in vivo* study from our group found selective effects of aripiprazole on the mesolimbic vs. the nigrostriatal dopaminergic pathways compared with haloperidol, which could be explained by the functional selectivity of aripiprazole [11]. However, how aripiprazole differentially affects the downstream signalling pathways of the D_2_R in various dopaminergic pathways *in vivo* has not been well studied.

Two major D_2_R downstream cellular signalling pathways have been identified. The canonical transduction pathway is the G protein-dependent cyclic adenosine monophosphate (cAMP)-protein kinase A (PKA) pathway, which mediates various cellular responses such as proliferation, metabolism and gene transcription [12–14]. Blockade of D_2_R in the brain is associated with the pharmacological properties of all antipsychotic drugs, including the therapeutic effects and some side-effects (e.g. EPS side-effects) [15]. However, whether and how D_2_R-mediated PKA signalling is involved in these pharmacological properties is not clear. Therefore, unveiling the effects of aripiprazole on the PKA signalling in various brain regions might elucidate the mechanism of aripiprazole and provide a new route for the development of improved antipsychotics. Furthermore, previous evidence suggested that D_2_-like dopamine autoreceptors have a preferential coupling to the PKA pathway to control dopamine synthesis [16, 17], release [18, 19] and uptake [20, 21]. However, previous studies showed discrepant influences of antipsychotic drugs on dopamine synthesis capacity. Acute treatment with haloperidol and aripiprazole both increased dopamine synthesis while quinpirole (a D_2_R agonist) reduced it in the caudate putamen (CPu) in rodents [22]. Additionally, acute administration with haloperidol in healthy human subjects induced a significant increase in dopamine synthesis capacity in various brain regions, including the striatum, mesencephalon, and medial prefrontal cortex (PFC) [23]. An *in vivo* study from our group demonstrated that aripiprazole down-regulated tyrosine hydroxylase (TH) mRNA expression in the ventral tegmental area (VTA), but not in the substantia nigra (SN), indicating reduced dopamine synthesis capacity after 1-week and 12-week treatment [11]. Therefore, the present study further investigated the relationship between the PKA activity and the TH activity after acute antipsychotic treatment.

Besides the canonical G protein-dependent cAMP-PKA signalling pathway, the non-canonical D_2_R transduction pathway is the G protein-independent protein kinase B (PKB/Akt)-glycogen synthase kinase 3 (GSK3) pathway. Studies implicated Akt and GSK3β signalling in the pathophysiology of neuropsychiatric disorders such as schizophrenia, bipolar disorder, and depression [24–26]. Studies on patients with schizophrenia showed decreased phosphorylation levels and GSK3 protein levels in the PFC [27–29]. In animal studies, GSK3β activity was elevated in the striatum, in a D_2_R- and β-arrestin2-dependent manner, under hyperdopaminergic conditions [24, 30–32]. An animal behavioural study comparing aripiprazole with β-arrestin2-biased D_2_R ligands also suggested that the β-arrestin2 signalling cascade can be simultaneously a significant contributor to antipsychotic therapeutic effects and a protective effect against motor side-effects [33]. Based on these findings, we proposed that aripiprazole might exert its therapeutic effects and reduced EPS side-effects via D_2_R-dependent Akt-GSK3β signalling.

Therefore, the present study examined the *in vivo* effects of aripiprazole on the cAMP-PKA and Akt-GSK3β signalling pathways in the mesolimbic, mesocortical and nigrostriatal dopaminergic pathways in comparison with a potent D_2_R antagonist—haloperidol and a D_2_R partial agonist—bifeprunox.

## Methods

### Animals and drug treatment

Male Sprague Dawley rats (aged 8 weeks) were obtained from the Animal Resource Centre (Perth, Australia). After arrival, all rats were divided into four treatment groups (*n* = 6/group). Rats were housed in individual cages under environmentally controlled conditions (temperature 22°C, light cycle from 07:00 to 19:00 h), with *ad libitum* access to water and standard laboratory chow diet. After 1-week acclimatisation to the new surroundings, all rats were injected with aripiprazole (0.75mg/kg, intraperitoneal (i.p.)), haloperidol (0.1mg/kg, i.p.), bifeprunox (0.8mg/kg, i.p.) or vehicle, respectively. All drugs were suspended in a 10% hydroxypropyl-β-cyclodextrin (Sigma, St. Louis, MO) solution. The dosages used in the present study were equivalent to the recommended dosage for treating schizophrenia patients, and calculated based on body surface area according to the FDA guidelines for clinical trials [34, 35]. Rats were euthanised by using carbon dioxide 2 hours after injection. This time-pointed was determined because all of the three drugs were rapidly absorbed after administration. For example, the plasma levels of haloperidol reached the maximum at 1 hour after oral and intramuscular administration in rats [36], while the aripiprazole concentration reached 80% of peak level in plasma and brain of rats at 1 hour after oral administration [37]. Although no pharmacokinetic data of bifeprunox were found in rats, a human study reported that bifeprunox reached peak plasma concentration within 1.5 hours after oral administration [38]. Brains were collected and then stored at -80°C. All rats were sacrificed between 10:00 A.M. and 12:00 A.M. to minimise possible circadian-induced variation of protein expression.

### Ethics Statement

All experimental procedures were approved by the Animal Ethics Committee (Application #: AE11/02), University of Wollongong, and complied with the Australian Code of Practice for the Care and Use of Animals for Scientific Purposes (2004). All animals were euthanised by using carbon dioxide. All efforts were made to minimise animal distress and prevent suffering.

### Microdissection

Following a standard procedure used in our lab, discrete brain regions were collected by using brain microdissection puncture [39–41]. Briefly, 500μm thickness fresh frozen brains were cut at -14°C and collected on glass slides. Based on the rat brain atlas [42], three sections through the forebrain (Bregma 3.30 to 4.20mm) were collected for microdissection of the PFC. Three sections through the striatum (Bregma 1.00 to 2.20mm) were collected for microdissection of the nucleus accumbens (NAc) and CPu. Three sections through the midbrain (Bregma -5.40 to -6.30mm) were collected for microdissection of the VTA and SN. For the PFC, NAc and CPu, bilateral punches (1.2mm) were collected from each of the three sections. For the VTA, each of the bilateral punches (0.8mm) was taken at a region medial to the medial lemniscus and dorsal to the interpeduncular nucleus. For the SN, each of the bilateral punches (0.5mm) was directed to the lateral border of the medial lemniscus. Tissue punches from each region were collected in microfuge tubes chilled on dry ice and kept frozen for future use.

### Western blot analyses

Tissue obtained from individual rat was homogenised and the supernatants were collected and stored at -80°C until required. Protein concentrations were determined spectrophotometrically at A750nm using the *DC* Protein Assay (Bio-Rad, #500–0111). Samples containing 10 μg of protein were resolved by 10% SDS–PAGE gels, and then transferred electrophoretically to a polyvinylidene difluoride (PVDF) membrane by using Bio-Rad Midi Format 1-D Electrophoresis Systems. The PVDF membranes were blocked for 1 hour at room temperature in Tris-buffered saline-Tween (TBST) containing 5% BSA, and incubated overnight at 4°C in primary antibodies diluted in TBST containing 1% BSA. Luminata Western horseradish peroxidase (HRP) Substrates (Millipore) and Kodak XBT-1 film were used to examine the membrane to visualise the immunoreactive bands. The immunoreactive signals were quantified using Bio-Rad Quantity One software. The data were then corrected based on their corresponding actin levels. All results were normalised by taking the value of the vehicle group as 100%. Experiments were performed in duplicate.

Four regulatory (RIα, RIIα, RIβ, and RIIβ) and two catalytic (Cα and Cβ) isoform genes have been identified previously [43]. PKA-RII expression is highest in brain tissues; PKA Cα and PKA Cβ are closely related (93% amino acid sequence similarity), PKA-Cα is ubiquitously expressed, and expression of PKA-Cβ is also highest in the brain [44–47]. Four antibodies for PKA subunits were chosen in the present study: PKA-RIIα (1:1000; Santa Cruz, #SC-908), PKA-Cα (1:1000; Cell Signalling, #5842), PKA-Cβ (1:1000; Santa Cruz, #SC-904) and phospho-PKA-C (Thr197) (1:1000; Cell Signalling, #5661). For the Akt-GSK3 pathway, the primary antibodies include: Akt (1:2000; Cell Signalling, #4691), phospho-Akt (Thr308) (1:1000; Cell Signalling, #13038), GSK3β (1:2000; Cell Signalling, #5676), and phospho-GSK3β (Ser9) (1:1000; Cell Signalling, #9322). We also examined tyrosine hydroxylase (1:1000; Millipore, #AB9983) and phospho-tyrosine hydroxylase (Ser40) (1:1000; Millipore #AB5935) in the VTA and SN to test their influence on dopamine synthesis. Mouse anti-actin primary polyclonal antibody (1:10000; Millipore, #MAB1501) and HRP-conjugated rabbit anti-mouse secondary antibody (1:3000; Cell Signalling, #7076) were used to determine the actin levels.

### cAMP measurement

The brain tissue was collected by microdissection described before, and assayed using a cAMP Direct Immunoassay Kit (Abcam, #ab65355) according to the manufacturer’s instructions. Briefly, the brain tissue was homogenised with 0.1M HCl, and centrifuged at 10,000g for 15 minutes at 4°C to obtain the supernatant. Then 100 μL of the diluted cAMP standard solution (0.039, 0.078, 0.156, 0.3125, 0.625, 1.25, 2.5, 5, and 10pmol/μl) or 50μl of the supernatant (added with 50μl 0.1M HCl) was mixed with kit reaction solution accordingly, and loaded to 96-well plates. All standards and samples were run in duplicate to ensure consistency of the reading. The plates were then treated with cAMP antibody, cAMP-HRP and HRP Developer, and measured spectrophotometrically at 450nm. The cAMP values obtained from the luminescence measurements were converted by the total protein amount determined using the *DC* Protein Assay (Bio-Rad, #500–0111) accordingly, expressed as picomole of cAMP per nanogram of protein.

### Statistics

All data was analysed using the SPSS Statistics v19.0 program. The data of both western blot analyses and cAMP measurement was normalised by taking the value of the control group as 100% and expressed as mean ± S.E.M. All phosphor proteins were also normalised to total protein levels. For example, p-PKA was normalised by the average levels of PKA-Cα and PKA-Cβ; p-TH, p-Akt and p-GSK3β were normalised by the levels of total TH, Akt and GSK3β, respectively. The Kolmogorov-Smirnov test was performed to test the normality of the data. One-way ANOVA was used if the data was normally distributed, followed by *Post Hoc* Tukey test to compare the control and drug treatment groups. Nonparametric Mann-Whiney U-test was applied when data was abnormal distributed. Statistical significance was accepted when *p* < 0.05.

## Results and Statistical Analyses

### The effects on the cAMP-PKA signalling pathway

#### PFC

**Fig 1:**
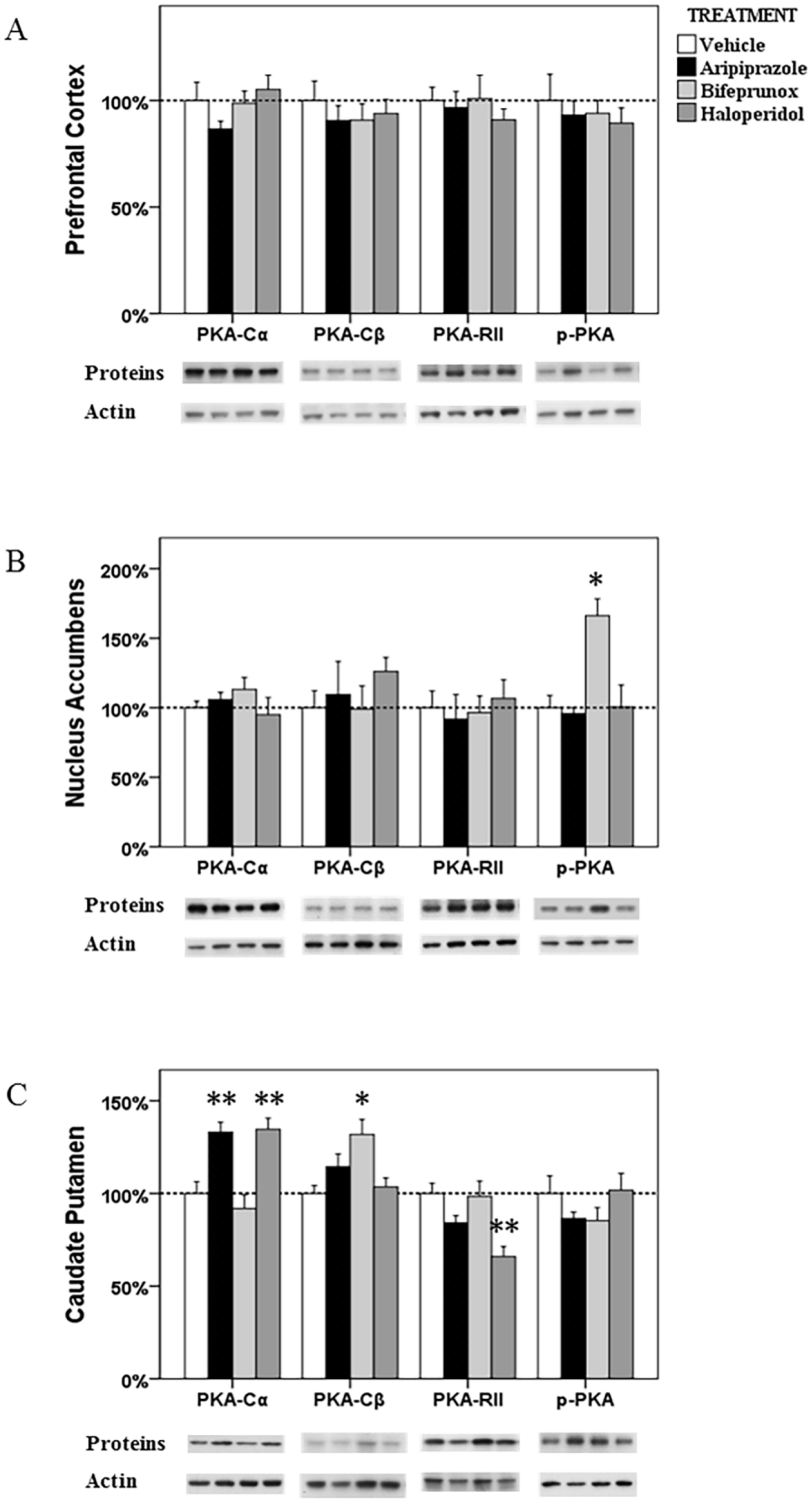
The alterations in the PKA signalling in the prefrontal cortex, nucleus accumbens and caudate putamen. The acute effects of three chemicals (aripiprazole, bifeprunox and haloperidol) on the protein levels of three PKA subunits (PKA-Cα,-Cβ, and-RII) and phospho-PKA in the prefrontal cortex (A), nucleus accumbens (B) and caudate putamen (C). (* *p* < 0.05, ** *p* < 0.01 *vs*. the control)

**Fig 2:**
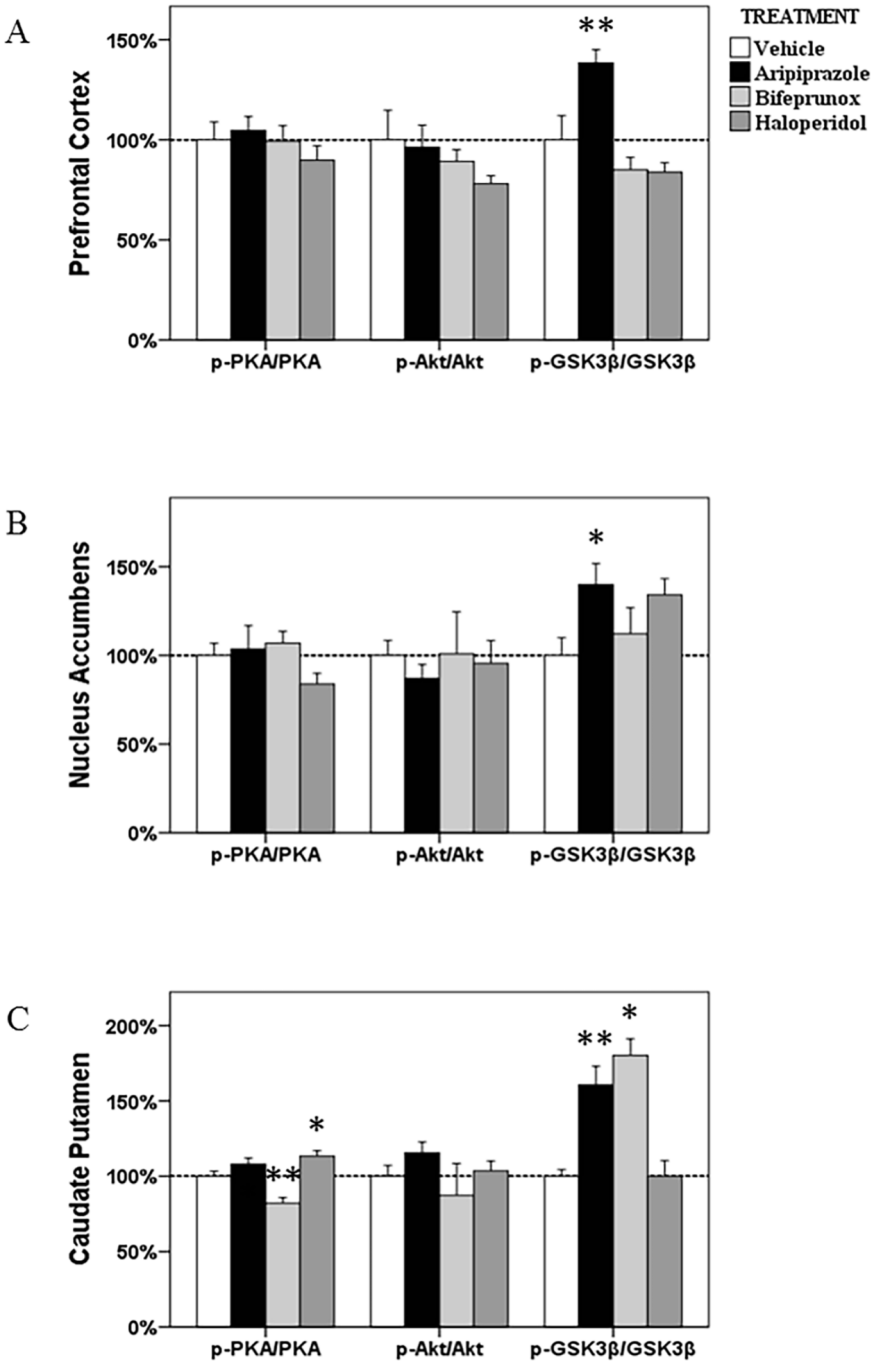
The ratios of p-Akt/Akt, p-GSK3β/GSK3β and p-PKA/PKA in the prefrontal cortex, nucleus accumbens and caudate putamen. The acute effects of three chemicals (aripiprazole, bifeprunox and haloperidol) on the ratios of p-Akt/Akt, p-GSK3β/GSK3β and p-PKA/PKA in the prefrontal cortex, nucleus accumbens and caudate putamen (* *p* < 0.05 *vs*. control; ** *p* < 0.01 *vs*. control).

ANOVA test did not identify any significant effects in the protein levels of PKA-Cα, PKA-Cβ, PKA-RII and p-PKA or the ratio of p-PKA/PKA after all three drug treatment in the PFC (Fig 1A and Fig 2A).

#### NAc

The levels of p-PKA in the NAc were shown to be significantly affected by drug treatment (*F*_3, 22_ = 11.157, *p* < 0.001); however, the ratio of p-PKA/PKA was also not significantly affected by drug treatment (*F*_3, 22_ = 1.390, *p* > 0.05). The protein levels of PKA-Cα, -Cβ, and-RII subunits did not change after drug treatment. *Post Hoc* analysis identified that only treatment with bifeprunox, not aripiprazole and haloperidol, enhanced the activity of the p-PKA subunit (+66.3%, *p* < 0.05) (Fig 1B).

#### CPu

ANOVA test revealed significant effects of drug treatment on the expression of PKA-Cα (*F*_3, 23_ = 11.806, *p* < 0.001), PKA-Cβ (*F*_3, 23_ = 4.985, *p* = 0.011) and PKA-RII (*F*_3, 21_ = 7.041, *p* = 0.002), but not p-PKA; however, the ratio of p-PKA/PKA was significantly changed by drug treatment (*F*_3, 23_ = 13.687, *p* < 0.001). *Post Hoc* analysis showed that both aripiprazole (*p* < 0.01) and haloperidol (*p* < 0.01) significantly elevated the expression of PKA-Cα, by 33.1% and 34.6%, respectively; only treatment with bifeprunox significantly increased the expression of the PKA-Cβ subunit by 31.9% (*p* < 0.05) (Fig 1C). Additionally, haloperidol treatment negatively affected expression of the PKA-RII subunit (-34.0%, *p* < 0.01). Moreover, both aripiprazole and haloperidol had no effects on the protein levels of PKA-Cβ; and bifeprunox did not alter the protein levels of PKA-Cα, and-RII subunits. The ratio of p-PKA/PKA was observed to be decreased by treatment with bifeprunox (*p* < 0.01), but increased by haloperidol treatment (*p* < 0.05); aripiprazole did not affect the ratio of p-PKA/PKA (Fig 2C).

#### VTA

**Fig 3:**
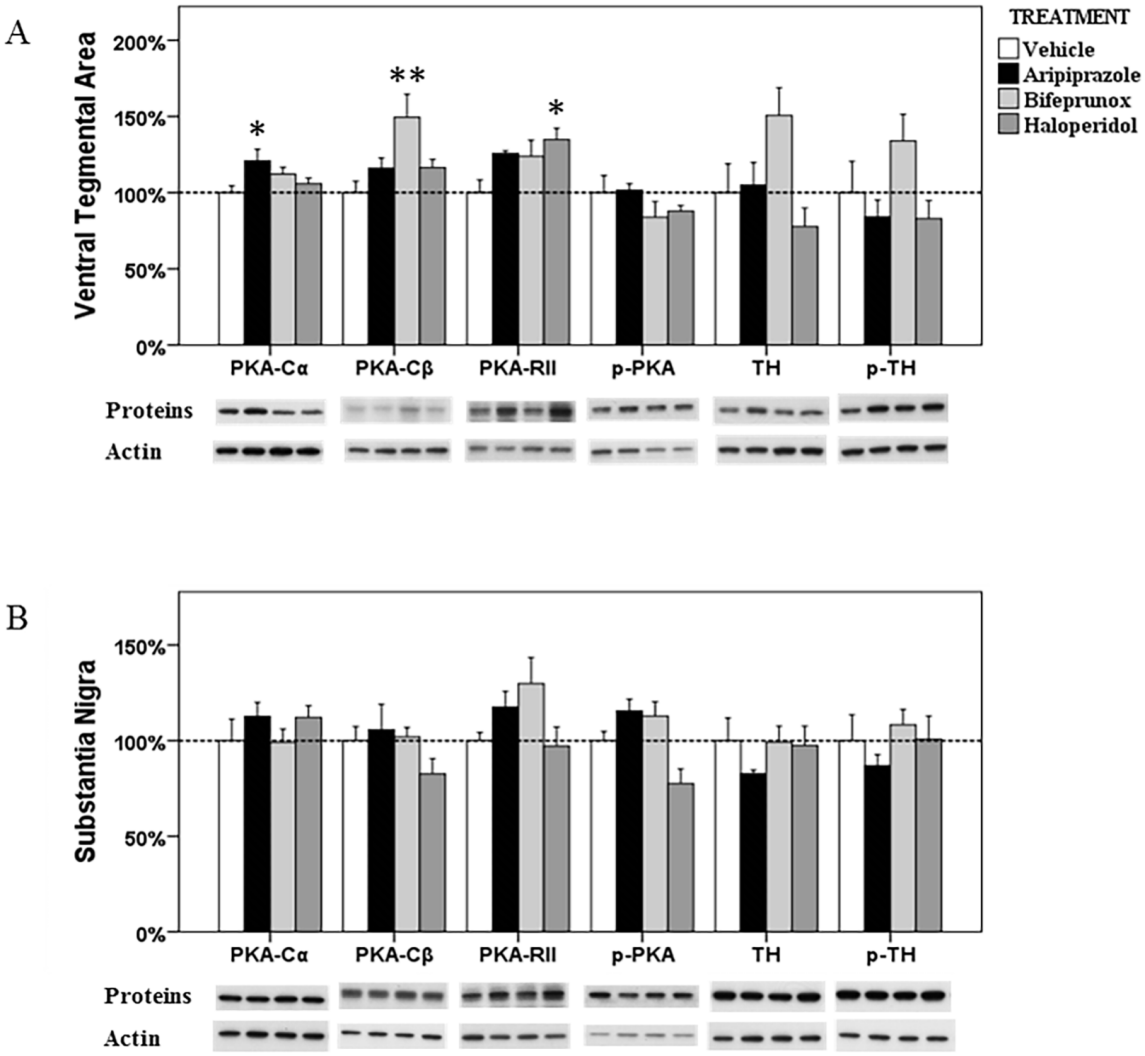
The alterations in the PKA signalling in the ventral tegmental area and substantia nigra. The acute effects of three chemicals (aripiprazole, bifeprunox and haloperidol) on the levels of three PKA subunits (PKA-Cα, -Cβ, and-RII), phospho-PKA, TH and phospho-PH in the ventral tegmental area (A) and substantia nigra (B). (* *p* < 0.05, ** *p* < 0.01 *vs*. the control)

**Fig 4:**
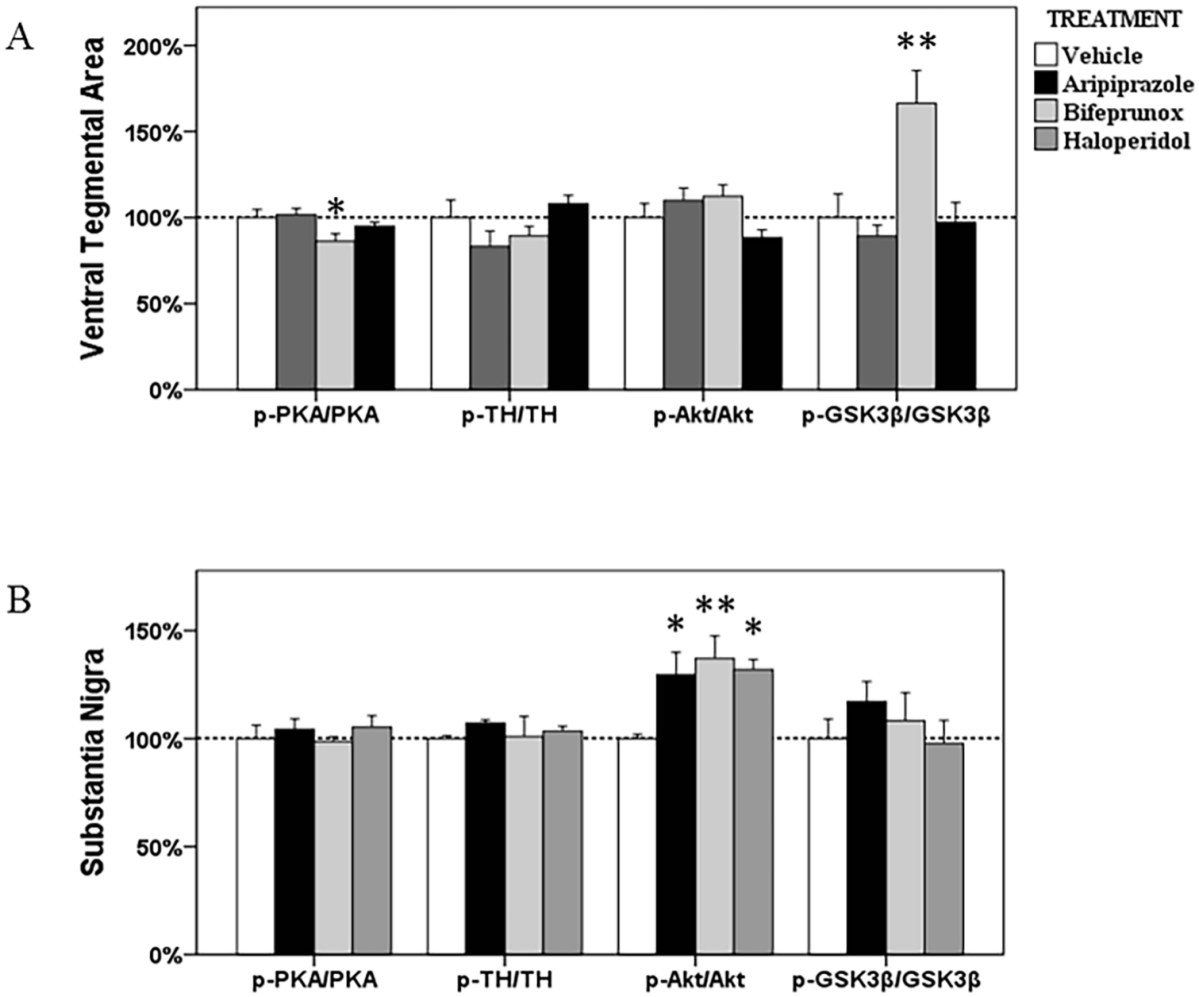
The ratios of p-Akt/Akt, p-GSK3β/GSK3β, p-PKA/PKA and p-TH/TH in the ventral tegmental area and substantia nigra. The acute effects of three chemicals (aripiprazole, bifeprunox and haloperidol) on the ratios of p-Akt/Akt, p-GSK3β/GSK3β, p-PKA/PKA and p-TH/TH in the ventral tegmental area and substantia nigra (* *p* < 0.05 vs. control; ** *p* < 0.01 vs. control).

The expression of PKA-Cα (*F*_3, 23_ = 3.757, *p* = 0.027), PKA-Cβ (*F*_3, 22_ = 5.079, *p* = 0.009) and PKA-RII (*F*_3, 23_ = 3.698, *p* = 0.029) has been significantly affected by drug treatment in the VTA. The ratio of p-PKA/PKA was also changed by drug treatment (*F*_3, 23_ = 3.197, *p* = 0.046), although the levels of p-PKA was not significantly altered (all *p* > 0.05). *Post Hoc* analysis demonstrated that the expression of both PKA-Cα and PKA-Cβ subunit was significantly increased by 20.9% and 49.4% by aripiprazole (*p* < 0.05) and bifeprunox (*p* < 0.01) treatment, respectively (Fig 3A). We also observed that bifeprunox treatment significantly reduced the ratio of p-PKA/PKA (*p* < 0.05) (Fig 4A). Additionally, treatment with haloperidol significantly elevated the expression of the PKA-RII subunit by 41% (*p* < 0.05). Furthermore, the levels of both TH and p-TH were showed to be increased by bifeprunox treatment, but not significantly (TH, +50.7%, *p* > 0.1; p-TH, +34.0%, *p* > 0.1); aripiprazole and haloperidol did not cause any noticeable change in the levels of TH and p-TH.

#### SN

ANOVA analysis identified significant effects of drug treatment on the levels of p-PKA (*F*_3, 19_ = 6.956, *p* = 0.003) drug treatment; however, *Post Hoc* analysis did not reveal any significant effects induced by any drug treatment (all *p* > 0.1) (Fig 3B). Additionally, the levels of PKA subunits, TH and p-TH were not altered by any drug treatment in the SN. The ratio of either p-PKA/PKA or p-TH/TH was not altered by any drug treatment (Fig 4B).

#### Measurement of cAMP

ANOVA analysis indicated significant effects on cAMP levels only in the PFC (*F*_3, 21_ = 3.529, *p* = 0.036). *Post Hoc* analysis identified that only bifeprunox significantly reduced the cAMP levels in the PFC (-53.3%, *p* < 0.05). There were no other notable changes in the cAMP levels in other four brain regions (all *p* > 0.05, Table 1).

**Table 1:** The cAMP levels (pmol/ng protein; mean values ± SEM; *n* = 6/group) in five brain regions after acute antipsychotic treatment.

	*PFC*	*NAc*	*CPu*	*VTA*	*SN*
**Aripiprazole**	75.0±16.6	55.0±7.9	34.3±7.5	166.7±32.7	85.0±13.3
**Bifeprunox**	49.0±7.4*	54.2±4.4	41.4±8.1	106.0±14.4	90.3±18.3
**Haloperidol**	74.8±15.2	58.9±3.2	40.7±3.5	115.8±8.3	132.5±22.5
**Control**	105.0±5.7	60.4±5.4	34.5±5.5	123.9±14.8	99.2±13.2

* *p* < 0.05

**Abbreviations:** CPu, caudate putamen; NAc, nucleus accumbens; PFC, prefrontal cortex; SN, substantia nigra; VTA, ventral tegmental area.

### The effects on the Akt-GSK3β pathway

#### PFC

**Fig 5:**
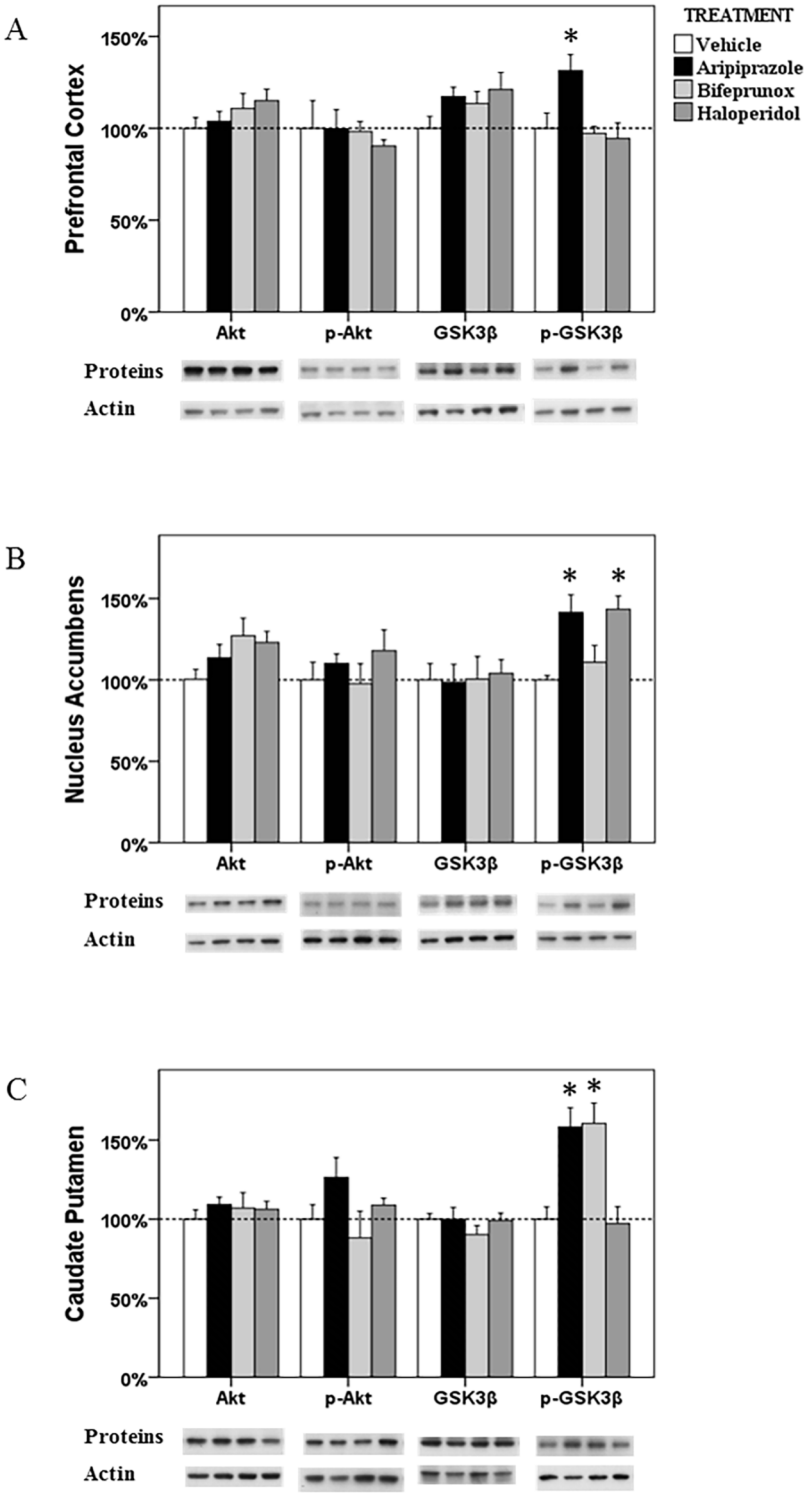
The alterations in the Akt-GSK3β in the prefrontal cortex, nucleus accumbens and caudate putamen. The acute effects of three chemicals (aripiprazole, bifeprunox and haloperidol) on the levels of Akt, phospho-Akt, GSK3β and phospho-GSK3β in the prefrontal cortex (A), nucleus accumbens (B) and caudate putamen (C). (* *p* < 0.05 *vs*. the control)

In the Akt-GSK3β signalling pathway, the levels of p-GSK3β was significantly influenced by drug treatment (*F*_3, 21_ = 7.504, *p* = 0.002) in the PFC; the ratio of p- GSK3β/GSK3β has also been significantly affected (*F*_3, 21_ = 10.435, *p* < 0.001). *Post Hoc* analysis indicated that only aripiprazole treatment, not haloperidol or bifeprunox treatment, significantly promoted the levels of p-GSK3β (+31.3%, *p* < 0.05) (Fig 5A). Aripiprazole also increased the ratio of p-GSK3β/GSK3β (*p* < 0.01) (Fig 2A).

#### NAc

ANOVA analysis showed that the levels of p-GSK3β were significantly affected by drug treatment (*F*_3, 23_ = 9.161, *p* = 0.001) in the NAc; ANOVA tests also revealed that drug treatment tended to significantly changed the ratio of p-GSK3β/GSK3β (*F*_3, 23_ = 2.585, *p* = 0.082). *Post Hoc* test revealed that the levels of p-GSK3β were significantly increased by both aripiprazole (*p* < 0.05) and haloperidol (*p* < 0.05), by 41.5% and 43.5%, respectively, whereas bifeprunox had no such effect (Fig 5B). In addition, the ratio of p-GSK3β/GSK3β was significantly increased by treatment with aripiprazole (*p* < 0.05); haloperidol treatment also tended to increase the ratio of p-GSK3β/GSK3β (*p* = 0.062) (Fig 2B).

#### CPu

Only the levels of p-GSK3β were shown to be significantly influenced (*F*_3, 22_ = 19.320, *p* < 0.001) by drug treatment in the CPu; the ratio of p-GSK3β/GSK3β was also significantly affected (*F*_3, 22_ = 17.103, *p* < 0.001). *Post Hoc* test identified significant enhancement on the levels of p-GSK3β induced by aripiprazole (+58.3%, *p* < 0.05) and bifeprunox (+60.6%, *p* < 0.05) treatment, respectively (Fig 5B). No drug treatment significantly influenced the levels of p-Akt, total Akt or total GSK3β. Furthermore, both aripiprazole and bifeprunox treatment significantly increased the ratio of p-GSK3β/GSK3β (aripiprazole, *p* < 0.01; bifeprunox, *p* < 0.05) (Fig 2C).

#### VTA

**Fig 6:**
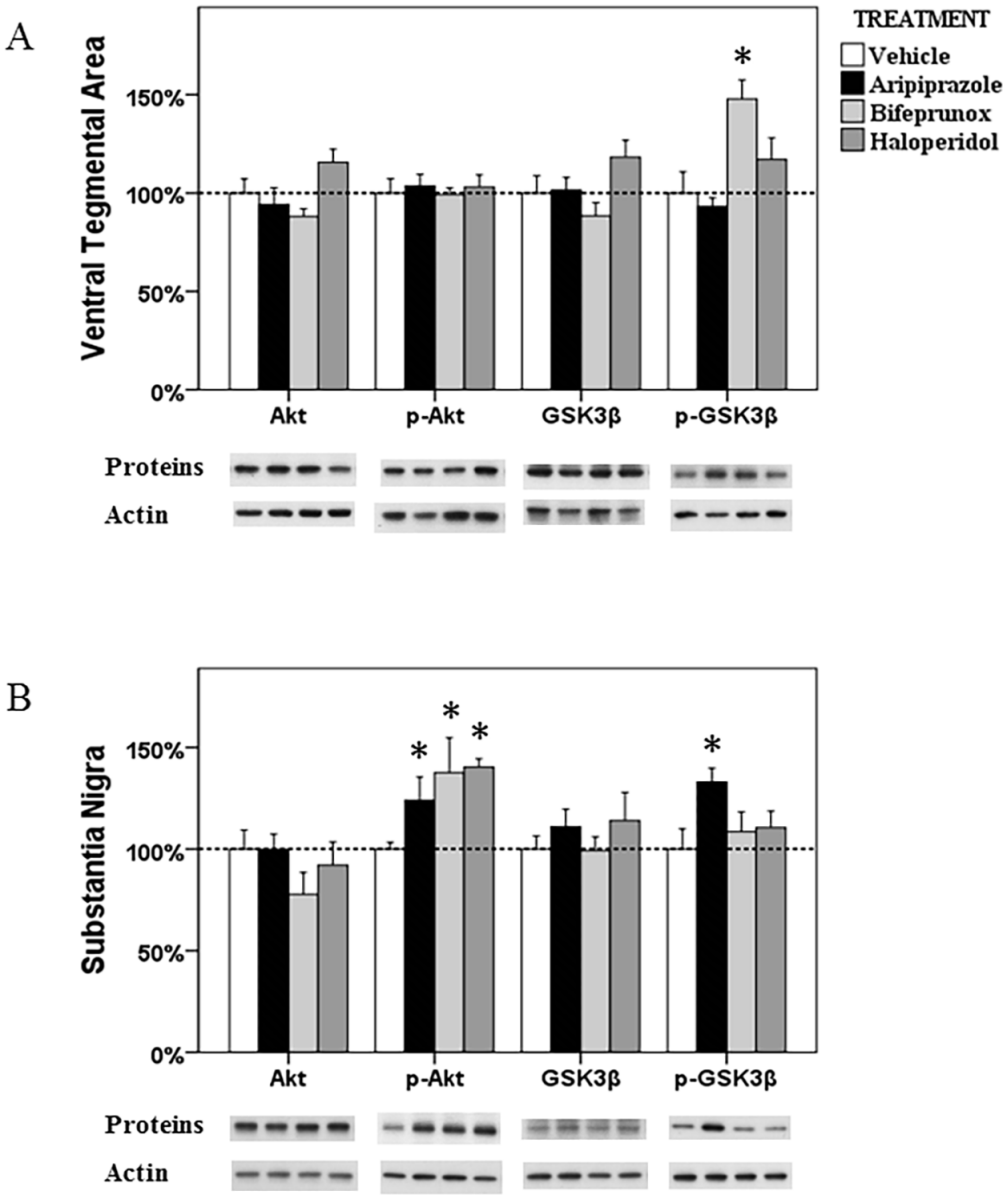
The alterations in the Akt-GSK3β in the ventral tegmental area and substantia nigra. The acute effects of three chemicals (aripiprazole, bifeprunox and haloperidol) on the levels of Akt, phospho-Akt, GSK3β and phospho-GSK3β in the ventral tegmental area (A) and substantia nigra (B). (* *p* < 0.05 *vs*. the control)

ANOVA analysis identified that only the levels of p-GSK3β in the Akt-GSK3β signalling pathway were significantly affected (*F*_3, 22_ = 7.124, *p* = 0.002) by drug treatment in the VTA; the ratio of p-GSK3β/GSK3β was also significantly changed (*F*_3, 22_ = 7.015, *p* = 0.002). *Post Hoc* test indicated a significant elevation in the expression of p-GSK3β after bifeprunox treatment (+47.8%, *p* < 0.05) (Fig 6A); the ratio of p-GSK3β/GSK3β was also significantly increased by bifeprunox (*p* < 0.01) (Fig 4A); no other significant result was observed.

#### SN

ANOVA test identified significant effects of drug treatment on the levels of p-Akt (*F*_3, 21_ = 4.243, *p* = 0.020) and the ratio of p-Akt/Akt (*F*_3, 21_ = 4.243, *p* = 0.020) in the SN; ANOVA analysis also indicated a trend towards significance on the levels of p-GSK3β (*F*_3, 20_ = 2.946, *p* = 0.061). *Post Hoc* analysis revealed that all three drugs significantly increased the levels of p-Akt (aripiprazole, +23.9%, *p* < 0.05; bifeprunox, +37.6%, *p* < 0.05; haloperidol, +40.3%, *p* < 0.05) (Fig 6B); they all elevated the ratio of p-Akt/Akt (aripiprazole, *p* < 0.05; bifeprunox, *p* < 0.01; haloperidol, *p* < 0.05) (Fig 4B). Additionally, only aripiprazole treatment significantly raised the levels of p-GSK3β by 33.0% (*p* < 0.05), but it did not significantly influence the ratio of p-GSK3β/GSK3β (*p* > 0.05).

## Discussion

The present study demonstrated the *in vivo* effects of aripiprazole on the downstream pathways of the D_2_R, by comparing it with haloperidol and bifeprunox. The results of the current study showed that aripiprazole had similar effects on PKA subunits to haloperidol, but not bifeprunox in the CPu and VTA, indicating that relatively lower intrinsic activity of aripiprazole on D_2_R might be the mechanism of aripiprazole to exert its therapeutic effects on treating positive symptoms of schizophrenia; on the other hand, aripiprazole displayed a very different action mode on the GSK3β activity from the other two chemicals, probably explaining its therapeutic effects on both positive and negative symptoms of schizophrenia, with reduced EPS. Together, these *in vivo* findings suggest that the relatively low intrinsic activity at D_2_R might be the reason that aripiprazole possesses unique pharmacological profiles and clinical effects from the other antipsychotic drugs.

### Antipsychotic effects on PKA signalling and TH activity

Dopamine is largely synthesised in the VTA and SN, and transported to the synapses in the PFC, CPu and NAc [48–51]. In the VTA and SN, short-form dopamine D_2_ autoreceptors are largely presented at pre-synapses, which generally provide a feedback mechanism that adjusts neuronal firing rate, dopamine synthesis capacity, and dopamine release in response to changes in extracellular dopamine level [52–54]. The G protein-dependent PKA signalling pathway is a canonical D_2_R downstream signalling pathway which mediates diverse cellular responses to external responses by activating the cAMP-dependent protein kinase including PKA kinase [12–14]. By reaction with cAMP, the inactive PKA could release a dimer of regulatory subunits, and two free monomeric catalytic subunits that can further phosphorylate other protein substrates [55]. It is suggested that the inhibition of the PKA transduction pathway induced by activation of D_2_ autoreceptors leads to reduced TH activity and decreased firing of dopamine neurons both *in vivo* and *in vitro* [56, 57]. In the present study, we observed that all three antipsychotics were able to differentially affect the expression of PKA subunits in various brain regions, as well as TH levels (although some results did not reach significance). For example, aripiprazole significantly increased the expression of PKA-Cα and PKA-RII subunits, and bifeprunox and haloperidol also significantly elevated the levels of PKA-Cβ and PKA-RII, respectively. In view of acute (2 hours) treatment, it is possible that these drugs may modulate protein expression of PKA subunits through affecting the actively translatable pool of mRNA, although further studies are necessary to confirm it. Moreover, aripiprazole reduced (not reaching significance) the expression of TH, while bifeprunox increased it (not significantly) in the VTA, which suggests that aripiprazole might potentially exert a different effect from that of bifeprunox and haloperidol on dopamine synthesis capacity. However, there were inconsistent reports about the effect of antipsychotics on dopamine synthesis capacity. For example, acute treatment with haloperidol and aripiprazole both increased dopamine synthesis while quinpirole (a D_2_R agonist) reduced it in rats CPu [22], although aripiprazole with agonistic activity at pre-synaptic D_2_ autoreceptors might reduce dopamine synthesis through feedback regulation [10, 58]. Moreover, acute administration with haloperidol in healthy human subjects induced a significant increase in dopamine synthesis capacity in various brain regions, including the striatum, mesencephalon, and medial PFC [23]. Our group using the same dosages of aripiprazole found that both 1-week and 12-week treatment with aripiprazole reduced the dopamine synthesis capacity in the VTA, but not in the SN [11]. The exact reason for the discrepancies between these studies remains unknown. One possible reason might be that the brain regions examined in the literature are not completely concordant with those in this study, which indicates that the effects of antipsychotics are quite brain-regional related; another possible reason might be the treatment dosages and treatment periods. For example, Der-Ghazarian’s study [22] used 10mg/kg for aripiprazole and 1mg/kg for haloperidol; these doses are considerably larger than those used in this study and might be a non-physiological response. It is worth noting that aripiprazole, in the present study, displayed different effects on the PKA pathway and TH activity from those of bifeprunox and haloperidol in the VTA and SN (although some changes did not reach significance), probably implying that D_2_R partial agonism or D_2_R antagonism cannot fully explain the unique pharmacological profile of aripiprazole. It is also worth pointing out that since the mesolimbic dopaminergic pathway is hyper-activated in schizophrenia, the increased dopamine synthesis capacity of bifeprunox in the VTA might partly explain why it failed to exert therapeutic effects in treating schizophrenia. The present study used only acute treatment, and we observed only some trends in the effects of antipsychotics (especially on the TH activity), possibly because the treatment period was too short. Therefore, experiments with longer treatment periods are required to confirm these findings.

In the PFC, CPu and NAc, long-form dopamine D_2_ receptors are highly expressed, regulating post-synaptic functions. Similar to the situation in the VTA and SN, these three drugs influenced the expression of PKA subunits in different manners in the PFC, CPu and NAc. Previous studies also suggested that aripiprazole inhibited cAMP accumulation and reduced PKA pathway signalling due to its partial agonism for the D_2_R, while haloperidol increased cAMP levels and facilitated its signalling [4, 8, 9, 59, 60]. Additionally, PKA-C levels were reported to be elevated in the CPu 15 minutes after acute treatment with haloperidol, but not the PFC and NAc [61]; the activity of the cAMP-PKA pathway in the striatum and protein levels of PKA-RII subunit in the striatum were also significantly increased after chronic treatment (21 days) with haloperidol due to its antagonism for the D_2_R [62]. In the present study, we observed that aripiprazole decreased PKA-Cα levels in the PFC, inhibiting PKA signalling, which is consistent with previous studies. Interestingly, our study exhibited that both aripiprazole and haloperidol affected PKA protein levels in a very similar pattern in the CPu and VTA; thereby, it is possible that both aripiprazole and haloperidol display similar antagonising effects on post-synaptic D_2_R in the CPu and VTA after acute treatment. Since aripiprazole is a D_2_R partial agonist, its intrinsic activity of aripiprazole for D_2_R is lower than that of endogenous dopamine, which might result in antagonising effects on D_2_R signalling; and this relatively low intrinsic activity might be the reason why not all D_2_R partial agonists could have meaningful therapeutic effects as aripiprazole. Lastly, only bifeprunox, a potent D_2_R partial agonist, was able to significantly inhibit the cAMP levels in the PFC, indicating that agonism for the D_2_R in the PFC might not contribute to the therapeutic effects of aripiprazole; hence, bifeprunox also displayed different effects on the PKA activity from both aripiprazole and haloperidol in the present study, which probably explains the failure of bifeprunox in clinic trials. The intrinsic activity might also play a critical role in the different effects between bifeprunox and aripiprazole. The intrinsic activity of bifeprunox for D_2_R is higher than that of aripiprazole [35]. At current dosage, the intrinsic activity of bifeprunox might not be low enough to induce antagonising effects as aripiprazole, although it is possible that the dosage of bifeprunox was insufficient to compete against endogenous dopamine to exhibit antagonising effects. This point can be verified by their different alterations in the ration of p-PKA/PKA in the CPu. The intrinsic activity of aripiprazole is neither high enough to positively affect the ratio of p-PKA/PKA as bifeprunox, nor low enough to negatively affect it as haloperidol. Since some D_2_R partial agonist with very low intrinsic activity (e.g. SDZ 208–912) could induce side-effects like EPS [63], a moderate intrinsic activity for the D_2_R might be a critical factor to develop optimum antipsychotics.

### Antipsychotic effects on the Akt-GSK3β signalling

In addition to the canonical G protein-dependent cAMP-PKA signalling pathway, the D_2_R signalling is also mediated by the Akt-GSK3β via β-arrestin2; many studies have indicated that Akt-GSK3β signalling plays a critical role in the pathophysiology of schizophrenia [24, 25, 27, 64]. Post-mortem studies on schizophrenic subjects demonstrated reduced phosphorylation levels and GSK3β protein levels in the PFC [28, 29]. In rodents, reduction of D_2_R- and β-arrestin2-dependent locomotor behaviours was observed in the situation of hyperactivity of GSK3β [24, 30–32].

The effects of antipsychotic drugs on Akt-GSK3β signalling have also been confirmed by a range of studies. *In vivo* studies indicated enhanced GSK3β phosphorylation in various brain areas of rodents after various first- and second-generation antipsychotic treatment [65–68]. A human study also suggested haloperidol treatment compensated for the decreased levels of endogenous Akt in the PFC in schizophrenic subjects, phosphorylating GSK3β, and leading to inhibition of its activity [69]. Collectively, antipsychotic drugs are capable of increasing the phosphorylation of GSK3β, thus inducing inhibition of the GSK3β kinase. The present study demonstrated that acute treatment with aripiprazole increased the levels of p-GSK3β, as well as the ratio of p-GSK3β/GSK3β in the PFC, CPu, NAc, respectively, which indicates the inhibition of the functions of GSK3β in these brain areas. On the other hand, bifeprunox elevated the levels of p-Akt in the SN and p-GSK3β in the CPu and VTA, as well as the ratios, simultaneously. Haloperidol reduced the activity of GSK3β by elevating the levels of p-GSK3β and the ratio of p-GSK3β/GSK3β in the NAc as well as increased the phosphorylation of Akt in the SN, but haloperidol did not affect GSK3β levels.

Obviously, the three compounds displayed distinct effects on Akt-GSK3β in the current study. Aripiprazole, in the present study, elevated the phosphorylation of GSK3β in the PFC, NAc, CPu and SN, thus resulting in inhibition of the activity of GSK3β in these brain regions. These findings suggested that aripiprazole probably exerted its therapeutic effects and reduced EPS by affecting GSK3β. A previous study found that acute treatment with various second-generation antipsychotics (including olanzapine, risperidone, clozapine) facilitated the phosphorylation of GSK3β in the cortex, striatum and hippocampus [68]. Thus, together with our findings, aripiprazole probably shares common mechanisms with other second-generation antipsychotics to act on the GSK3β signalling to exert therapeutic effects. On the other hand, bifeprunox had fewer effects on GSK3β than aripiprazole, especially in the PFC and NAc. The reasons that aripiprazole and bifeprunox showed different effects on GSK3β signalling could be their different intrinsic activities at D_2_R, however we could not completely exclude the possibility that the dosage of bifeprunox used in this study was insufficient to act on GSK3β signalling. Haloperidol here displayed very limited effects on GSK3β, which might indicate that haloperidol does not exert therapeutic effects via GSK3β signalling. Moreover, a previous study testing animal behaviour after treating β-arrestin2-biased D_2_R ligands along with aripiprazole indicated that activation of β-arrestin2 contributed to the protection against motor side-effects [33]. Whether inhibition of GSK3β followed by activation of β-arrestin2 is directly linked to this protective effect is not clear. However, our finding that aripiprazole, but not haloperidol, reduced the activity of GSK3β in the CPu might provide an explanation. Lastly, it is worth noting that the total levels of GSK3β did not change after acute treatment with all three compounds, which is consistent with the findings of Alimohamad’s studies [65, 66].

It is clearly seen that the phosphorylation of GSK3β did not change completely in the same manner as that of Akt in the present study. Two phosphorylating sites of Akt have been identified: Thr308 and Ser473, both of which can be affected by antipsychotics [24, 31, 32, 70]. In the current study, the Thr308 site was examined since phospho-Thr308-Akt was indicated to be involved in the β-arrestin2/Akt/PP2A complex associated with D_2_R [31, 32], but not phospho-Ser473-Akt. However, Akt with either of the sites phosphorylating can induce phosphorylation of GSK3β at Ser9 that was examined in this study. Thus, it is very possible that the increased levels of p-GSK3β in the present study might be induced by either phospho-Thr308- or phospho-Ser473-Akt from different signalling pathways. Although an animal behavioural study revealed that β-arrestin2-associated GSK3β signalling in D2R-expressing neurons is essential for the antipsychotic effects of aripiprazole [71], the results of the present study suggested that these drugs might affect the activities of GSK3β via multiple signalling pathways. In addition to the modulation of the β-arrestin2-mediated signalling pathway, GSK3β is also involved in the wingless (Wnt)-dishevelled-3 (Dvl3)-β-catenin signalling pathway [72]. Kang and Sutton found that both haloperidol and clozapine regulate the activity of GSK3β through Wnt signal pathways involving Dvl upstream in SH-SY5Y cells [73, 74]. *In vivo* studies indicated that haloperidol treatment significantly increased the phosphorylation of GSK3β at Ser9 in various brain regions through Wnt-Dvl3-β-catenin signalling [65, 66]. Another report found that aripiprazole was also able to increase the phosphorylated GSK3β along with elevated β-catenin levels *in vivo* [75]. Moreover, aripiprazole can also act at 5-HT_1A_ receptors which also targets GSK3β [76]. It has been reported that activation of 5-HT_1A_ receptors led to increased phosphorylation of GSK3β *in vivo* [77]. Evidence also indicated that aripiprazole, at the same dose as the present study, significantly increased 5-HT_1A_ receptor binding density *in vivo* [78]. However, further studies are required to investigate whether 5-HT-regulated GSK3β signalling is involved in the pharmacological properties of aripiprazole. Taken together, the results of the present study suggested that regulation via β-arrestin2/Akt/PP2A signalling pathway by antipsychotics might partly contribute to the alterations in the activities of GSK3β; and the alterations in the activities of GSK3β we observed in the present study might be an integrated consequence of multiply signalling pathways regulated by antipsychotics.

### Comparison between aripiprazole and haloperidol and bifeprunox

#### Aripiprazole vs. haloperidol

Haloperidol is a potent D_2_R antagonist [76], treating positive symptoms of schizophrenia effectively, but also inducing severe EPS. In the present study, aripiprazole and haloperidol displayed, to some extent, similar effects on PKA signalling in the mesolimbic dopaminergic pathway, indicating that aripiprazole might perform as an antagonist in the mesolimbic dopaminergic pathway within, at least, a few hours after treatment. In contrast, aripiprazole showed much stronger effects on the levels of p-GSK3β in several brain regions (e.g. PFC, NAc), probably elucidating haloperidol’s inability to treat negative symptoms of schizophrenia, since acute treatment with some other second-generation antipsychotics were able to widely affect GSK3β [68]. Furthermore, since activation of β-arrestin2 contributes to the protective effects against motor side-effects [33], as discussed before, the effects of aripiprazole in the CPu, compared with haloperidol, might reveal a connection between the inhibition of GSK3β activity and reduced EPS.

#### Aripiprazole vs. bifeprunox

Bifeprunox is a D_2_R partial agonist that failed in clinic trials. Some researchers have suggested that the therapeutic effects of aripiprazole are attributable to its partial agonism at the D_2_R [79, 80]. In the present *in vivo* study, it is obvious that bifeprunox affected both PKA and Akt-GSK3β transduction pathways and TH activity in a very different manner from aripiprazole. The action mode of aripiprazole on GSK3β is more like that of the second-generation antipsychotic drugs, not bifeprunox, probably due to a relatively lower intrinsic activity of aripiprazole at D_2_R. However, we could not completely exclude the possibility that the dosage of bifeprunox used in the present study was insufficient to induce antagonising effects on D_2_ receptors.

In the present study, by comparison with haloperidol and bifeprunox, neither antagonism nor partial agonism for the D_2_R could completely explain the unique activities of aripiprazole on the D_2_R downstream signalling pathways. It is worth noting that functional selectivity has been proposed to explain the unique pharmacological properties of aripiprazole [1]. Unfortunately, no evidence in the present study can directly support the theory of functional selectivity for aripiprazole. It is probably because the treatment in the present study was too short to take effect; and the *in vivo* cellular environment is very complex and the activities of neurons in the living brain are regulated by multiple and convergent factors. Therefore, long-term study and more research methods are required to study this issue.

It is worth pointing out that acute antipsychotic treatment exerted very few or no effects on the levels of p-PKA and cAMP levels in this study. Beaulieu et al. stated that within the first 30min after activation of D_2_R, G protein-coupled signalling induced a rapid and transient change in the PKA transduction pathway, resulting in a short-term response; after 30min, the Akt-GSK3 transduction pathway was activated, resulting in a more progressive and longer-lasting response [81]. In this study, we sacrificed animals 2 hours after drug administration. Therefore, it is possible that the activation of the PKA signalling pathway had already diminished when we sacrificed animals and collected brains.

There were also some limitations in the present study. It is worthy to note that the signalling pathways examined in the present study were multi-targeted, and the drugs could react with other dopamine receptors other than D_2_R. For example, the three compounds used in this study have affinities with dopamine D_3_ receptors. Therefore, we cannot completely exclude the effects of other dopamine receptors; further experiments are important to investigate the effects of antipsychotics on these signalling pathways through specific subtypes of dopamine receptors. Another limitation of the present study is that only the changes in the protein levels of the signalling pathways were examined, further studies are necessary to examine the functional and behavioural changes followed treatment of these antipsychotic drugs.

## Conclusions

The present study demonstrated the various effects of acute treatment with aripiprazole, bifeprunox and haloperidol on the two downstream signalling pathways of the D_2_R in five brain regions. Our study revealed that acute treatment of aripiprazole had differential effects on both cAMP-PKA and Akt-GSK3β signalling pathways from haloperidol and bifeprunox in various brain areas. Furthermore, the differential acute effects of aripiprazole (compared with haloperidol) on GSK3β signalling were also observed in the current study. Further studies in the effects of chronic aripiprazole treatment on these signalling pathways in a schizophrenia animal model might help to explain why aripiprazole has therapeutic effects on negative symptoms of schizophrenia, along with reduced EPS. Our study also suggested that the unique pharmacological profile of aripiprazole might contribute to the relatively low intrinsic activity at the dopamine D_2_ receptor. Further studies are required to explore the involvement of functional selectivity theory in the mechanisms of aripiprazole.
